# The potential role of *Listeria monocytogenes* in promoting colorectal adenocarcinoma tumorigenic process

**DOI:** 10.1186/s12866-024-03240-5

**Published:** 2024-03-15

**Authors:** Giulia Baldelli, Mauro De Santi, Collins Njie Ateba, Giorgia Cifola, Giulia Amagliani, Christ-Donald Kaptchouang Tchatchouang, Peter Kotsoana Montso, Giorgio Brandi, Giuditta Fiorella Schiavano

**Affiliations:** 1 Department of Biomolecular Sciences, University of Urbino Carlo Bo, Urbino (PU), Urbino, Italy; 2 Food Security and Safety Focus Area, Faculty of Natural and Agricultural Sciences, North-West University, Mmabatho, South Africa; 3 Department of Humanities, University of Urbino Carlo Bo, Urbino (PU), Urbino, Italy

**Keywords:** *Listeria monocytogenes*, Colorectal cancer, Tumorigenic process

## Abstract

**Background:**

*Listeria monocytogenes* is a foodborne pathogen, which can cause a severe illness, especially in people with a weakened immune system or comorbidities. The interactions between host and pathogens and between pathogens and tumor cells have been debated in recent years. However, it is still unclear how bacteria can interact with tumor cells, and if this interaction can affect tumor progression and therapy.

**Methods:**

In this study, we evaluated the involvement of *L. monocytogenes* in pre-neoplastic and colorectal cancer cell proliferation and tumorigenic potential.

**Results:**

Our findings showed that the interaction between heat-killed *L. monocytogenes* and pre-neoplastic or colorectal cancer cells led to a proliferative induction; furthermore, by using a three-dimensional cell culture model, the obtained data indicated that *L. monocytogenes* was able to increase the tumorigenic potential of both pre-neoplastic and colorectal cancer cells. The observed effects were then confirmed as *L. monocytogenes*-specific, using *Listeria innocua* as negative control. Lastly, data suggested the Insulin Growth Factor 1 Receptor (IGF1R) cascade as one of the possible mechanisms involved in the effects induced by *L. monocytogenes* in the human colorectal adenocarcinoma cell line.

**Conclusions:**

These findings, although preliminary, suggest that the presence of pathogenic bacterial cells in the tumor niches may directly induce, increase, and stimulate tumor progression.

**Supplementary Information:**

The online version contains supplementary material available at 10.1186/s12866-024-03240-5.

## Introduction

Colorectal cancer (CRC) is the third most common cause of cancer mortality worldwide; moreover, a recent report has predicted about 3.2 million cases of colorectal cancer in 2040, based on the projection of aging, population growth, and human development [1]. The incidence results are approximately 4 times higher in developed countries in comparison with developing ones; this different trend is mainly due to the direct relation between CRC incidence and the human development index, which considers life expectancy, education (lifestyle and diet changes), and gross national income. This aspect makes CRC a marker of socioeconomic development [2].

The colon is considered the natural niche for several competitive bacteria; dysbiosis in the intestinal microbiome could induce chronic inflammation, carcinogenic metabolite production, and neoplasia induction [3, 4]. Risk factors for CRC include genetic predisposition, age, obesity, exposure to chemicals or radiations, and behavioral factors such as physical inactivity, smoking, and alcohol consumption; moreover, the potential role of bacterial infections as carcinogen factors and cancer promoters is now gaining a great interest [5, 6].

Several mechanisms are known to be involved in bacterial-induced cancer development, through host signaling pathways modulation, toxins production with further DNA damage, affecting cell proliferation and death, or immune response and signaling [7–9]. Furthermore, different pathways have been described as possibly implicated in CRC formation and carcinogenesis, as the bacteria-produced pro-inflammatory cytokines and stimulation of signaling mechanisms involved in tumor progression, the bacteria-driven immune modulation, and the presence of virulence factors or the production of toxic metabolites [10–14].

*Listeria monocytogenes* is a pathogenic bacterium, listeriosis-causing. This pathology is a foodborne disease, with a low incidence (0.46 in 100,000 people in Europe), but with a very high fatality rate (∼ 18%), compared with other foodborne diseases [15, 16].

Furthermore, different authors have demonstrated that some strains of *L. monocytogenes* can alter the host intestinal microbiome, promoting further intestinal bacterial colonization and deeper organ infection [17, 18].

While only one bacterium, *Helicobacter pylori*, has so far been included in the International Agency for Research on Cancer (IARC)’s list of carcinogenic pathogens [19], many other bacteria have been discovered to have carcinogenic effects [5]. *L. monocytogenes* induces hepatocarcinoma proliferation in vivo through the activation of mitogen-activated protein kinases and nuclear factor-KB in tumor cells. The same authors showed comparable data, obtained by in vitro experiments treating hepatocarcinoma cells with heat-killed (HK) *L. monocytogenes* [20].

In agreement with these data, similar proliferative stimulating effects were observed with *H. pylori*, known to be a possible responsible for gastric cancer initiation through the induced increase of Insulin Growth Factor 1 Receptor (IGF1R) expression [20, 21].

To our knowledge, no data are available about the possible involvement of *L. monocytogenes* in initiating and promoting the CRC tumorigenic process. Therefore, in this preliminary study, the capacity of *L. monocytogenes* to enhance proliferative and tumorigenic potentials in a pre-neoplastic and a colorectal cancer cell line was assessed, evaluating the modulation of the IGF1R molecular pathway, too.

## Materials and methods

### Cell cultures

The pre-neoplastic murine skin epidermal JB6 Cl 41-5a promotion sensitive (JB6 P+) cells and CaCo2 cells, a human colorectal adenocarcinoma cell line, were purchased from the American Type Cell Culture Collection (ATCC, Rockville, MD, USA). Cells were cultured in Eagle’s Minimal Essential Medium (EMEM), supplemented with 15% or 5% heat-inactivated fetal bovine serum (FBS) (CaCo2 cells or JB6 P + cells, respectively), 2mM L-glutamine, 1x MEM Non-Essential Amino Acid Solution, 0.1 mg/mL streptomycin, 0.1 U/L penicillin, and 1mM Na-pyruvate.

For the JB6 P + cells, the 12-O-tetradecanoylphorbol-13-acetate (TPA) was used as a tumor promoter and dissolved in dimethyl sulfoxide (20 ng/mL). All cell culture materials were purchased from Sigma-Aldrich (St. Louis, MO, USA). The cells were grown in a humidified atmosphere at 37 °C with 5% CO_2_.

### Bacterial strains

*L. monocytogenes* 1484 selected for this study was isolated from ready-to-eat (RTE) food samples and Whole-Genome Sequenced, as described by Schiavano et al. [22]. *Listeria innocua* ATCC33090 was used as negative control. *L. monocytogenes* and *L. innocua* were grown on *Agar Listeria according to Ottaviani & Agosti* (ALOA) plates, according to ISO 11290-1-2: 2017 [23], and incubated aerobically at 32 °C for 24 h and then subcultured in tryptic soy yeast extract agar (TSYEA) plates. Overnight cultures of the bacterial strains were grown in tryptic soy yeast extract broth (TSYEB) at 32 °C, and were washed and resuspended in cells’ culture medium (EMEM), then adjusted at optical densities at 600 nm (OD_600_) corresponding to a final concentration of 1 × 10^8^ CFU/ml. The bacterial suspensions’ concentration was experimentally confirmed by plate culture, at each experiment. All culture materials were purchased by Thermo Fisher Scientific (Waltham, MA USA).

### Bacterial strains’ heat-inactivation

*L. monocytogenes* and *L. innocua* were inactivated following a heating step, placing bacterial suspensions in a water bath at 70 °C for 1 h. The complete inactivation of the bacteria was confirmed by plating 100 µl of the HK suspensions in a TSYEA plate and incubating overnight at 32 °C.

### Cell proliferation assay

JB6 P + cells (1 × 10^5^ cells/well) or CaCo2 cells (8 × 10^4^ cells/well) were seeded in 24 well or 6-well plates respectively, and incubated for 48 h, at 37 °C and 5% CO_2_. Then, the culture medium was replaced with EMEM (in the control dishes), suspensions of HK *L. monocytogenes* (1:10, 1:20 or 1:50 cell-to-HK bacteria ratios), or suspensions of HK *L. innocua* (for negative control dishes, 1:10, 1:20 and 1:50 cell-to-HK bacteria ratios) and incubated for 48 or 72 h (CaCo2 and JB6 P+, respectively). As a further negative control, suspensions of inert latex beads were also used, at the same ratios as bacterial suspensions. Moreover, for the JB6 P + cells, TPA was utilized as a tumor promoter, as positive control. Cell viability was evaluated with two different methods, performing the cell count and MTS assays, as previously reported [24, 25]. Briefly, for the cell count method, viable and dead cells were counted by trypsinization and using a hemocytometer, by trypan blue exclusion assay. Moreover, also the CellTiter 96® AQueous Non-Radioactive Cell Proliferation Assay was used to determine the number of viable cells.

### Anchorage-independent transformation assay (soft agar assay)

CaCo2 or JB6 P + cells were cultured in soft agar with 15% v/v FBS, as previously reported [25]. Briefly, 2 × 10^3^ CaCo2 were cultured in 12 well plates, resuspending them in an agar layer composed of 0.3% v/v agar-15% FBS-EMEM and cells (CTR), or supplemented by 1:10, 1:20 or 1:50 cell-to-HK *L. monocytogenes*, cell-to-HK *L. innocua* ratios, or cell-to-inert latex beads ratios and maintained at 37 °C, 5% CO_2_ for 28–31 days. The same experimental conditions were tested for JB6 P + cells (5 × 10^3^ cells/well); for this cell line, TPA was also tested as a positive control, as a tumor promoter. After the incubation period, the cells were stained with 0.01% w/v crystal violet, counted, and photographed with a stereoscope. Only 3D colonies formed by more than 20 cells were considered.

### Western immunoblot

CaCo2 cells were seeded in 35 mm culture dishes (1.6 × 10^5^ cells/dish) and incubated for 48 h. Then, the culture medium was replaced with EMEM (in the control dishes), suspensions of HK *L. monocytogenes* (1:10, 1:20, or 1:50 cell-to-HK bacteria ratios), or suspensions of HK *L. innocua* and inert latex beads (for negative control dishes, 1:10, 1:20 and 1:50 cell-to-HK bacteria ratios or cell-to-latex beads ratios), and cells were lysed after 30 or 60 min. Furthermore, samples of HK *L. monocytogenes* and HK *L. innocua* were lysed with the same buffer and loaded into the gel to confirm that the generated chemiluminescent signal was not given by HK bacteria.

The cellular protein expression and phosphorylation were analyzed by western blotting, as previously reported [26]. Briefly, cells were lysed on ice with 20 mmol/L HEPES (pH 7.9), 0.42 mol/L NaCl, 25% v/v glycerol, 0.2 mmol/L EDTA, 0.5%v/v Nonidet P-40, 1.5 mmol/L MgCl_2_, 1 mmol/L NaF, 1 mmol/L Na_3_VO_4_, and 1× complete protease inhibitor cocktail (Roche Diagnostics Ltd., Mannheim, Germany). After two freezing and thawing steps, a clarification phase was performed by centrifugation at 12,000 rpm for 10 min at 4 °C; the total cell lysates were quantified by Bradford assay (Sigma-Aldrich, St. Louis, Missouri, USA) and then fractionated by SDS-PAGE, and transferred to a nitrocellulose membrane (0.2 μm pore size) (Bio-Rad Laboratories Inc., Hercules, CA, USA). The following primary antibodies have been used: phospho-IGF-1 Receptor β (Tyr1135/1136) (#3024), IGF-1 Receptor β (#3027), phospho-p44/42 MAPK (ERK1/2) (Thr202/Tyr204) (#9101), p44/42 MAPK (ERK1/2) (9102), purchased from Cell Signaling Technology (Beverly, MA, USA). Horseradish peroxidase-conjugated secondary antibodies were used to detect protein bands (Bio-Rad Laboratories Inc). The obtained blots were incubated with enhanced chemiluminescence reagents (Clarity Western ECL Substrate, Bio-Rad, Hercules, California, USA), and the ChemiDoc Imager was used to detect the immunoreactive bands (Bio-Rad Laboratories Inc). The resulting blots were exported and quantified with Image Lab Software (Bio-Rad Laboratories Inc).

### Statistical analyses

Statistical analyses were performed using one-way ANOVA, followed by Dunnett’s Multiple Comparison Test, using the GraphPad Software. A p-value lower than 0.05 was considered significant.

## Results

### Heat-killed *L. monocytogenes* modulates proliferation and tumorigenic transformation capacity of pre-neoplastic cells

To evaluate the involvement of *L. monocytogenes* in tumor progression, the proliferative capacity of a pre-neoplastic cell line (JB6 P+) was assessed after the interaction with suspensions of different cell-to-bacteria ratios (1:10, 1:20, or 1:50). The bacterial suspensions were inactivated with a thermal treatment, to avoid the difficulties linked to the use of a living pathogen in the in vitro long-lasting experimental models used, as the damages induced by whole highly replicative living bacteria on eukaryotic cells.

**Fig. 1:**
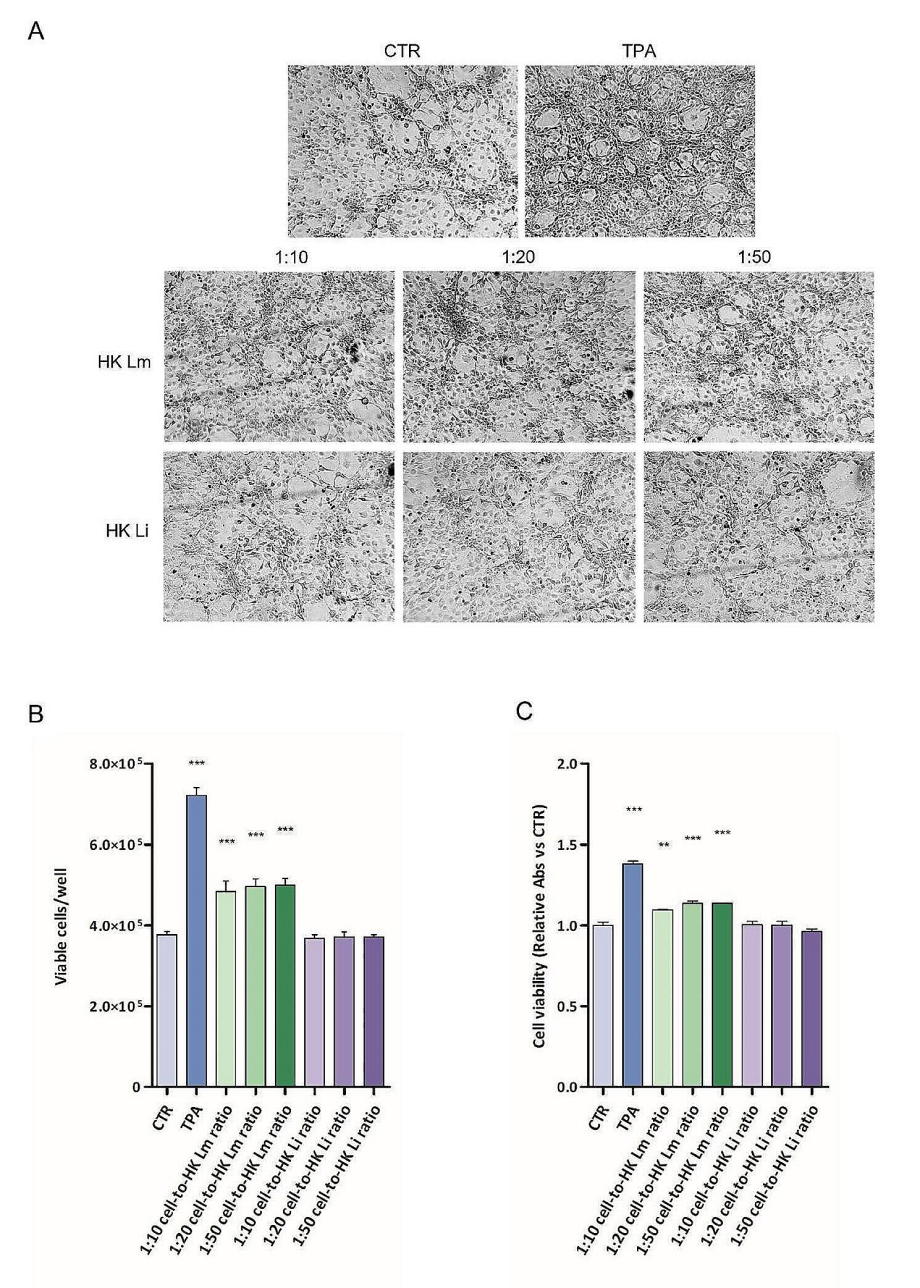
Effects of HK *L. monocytogenes* on JB6 P + cell transformation and proliferation in anchorage-dependent conditions. Cells were cultured in contact with 1:10, 1:20, or 1:50 cell-to-HK *L. monocytogenes* ratios, or with 1:10, 1:20, or 1:50 cell-to-HK *L. innocua* ratios, for 72 h. Representative pictures of the results (A). Cell viability was assessed through cell count by trypan blue exclusion assay (B) and MTS assay (C). Data show results of at least three different experiments; results are expressed as the total number of cells ± standard error. Statistical significance is compared to CTR. *** *p* < 0.001; one-way ANOVA, followed by Dunnett’s Multiple Comparison Test. TPA: tumor promoter; HK Lm: heat-killed *L. monocytogenes*; HK Li: heat-killed *L. innocua*

Representative pictures of the results are reported in Fig. 1A. A 72-hour HK *L. monocytogenes* treatment of pre-neoplastic JB6 P + cells strongly enhanced cell transformation and proliferation capacity. In contrast, data obtained after 72 h of contact between cells and *HK L. innocua* or between cells and latex beads did not change the transformation and proliferation capacity of the pre-neoplastic cell line, resulting in a number of viable cells comparable to the control one. The TPA transformation induction was confirmed, resulting in a 1.91-fold increase in viable cell number, in comparison to the control. Moreover, a statistically significant increase in cell proliferation was also obtained after cell interaction with all cell-to-HK *L. monocytogenes* ratios tested (1:10 cell-to-HK *L. monocytogenes* ratio: 1.28 ± 0.07; 1:20 cell-to-HK *L. monocytogenes* ratio: 1.32 ± 0.05; 1:50 cell-to-HK *L. monocytogenes* ratio: 1.32 ± 0.04). Conversely, the proliferation rates obtained with 1:10, 1:20, and 1:50 cell-to-HK *L. innocua* ratios were not affected in comparison to the control (fold changes: 0.98 ± 0.02, 0.99 ± 0.03, 0.98 ± 0.02, respectively) (Fig. 1B). The same trend of cell viability results was achieved with MTS assay (Fig. 1C).

Comparable results were obtained by the inert latex beads suspensions, with no change in cell viability after 72 h of contact (Fig. S1). Moreover, the mortality rate of cells was not affected either by TPA or bacterial suspensions (Fig. S2).

**Fig. 2:**
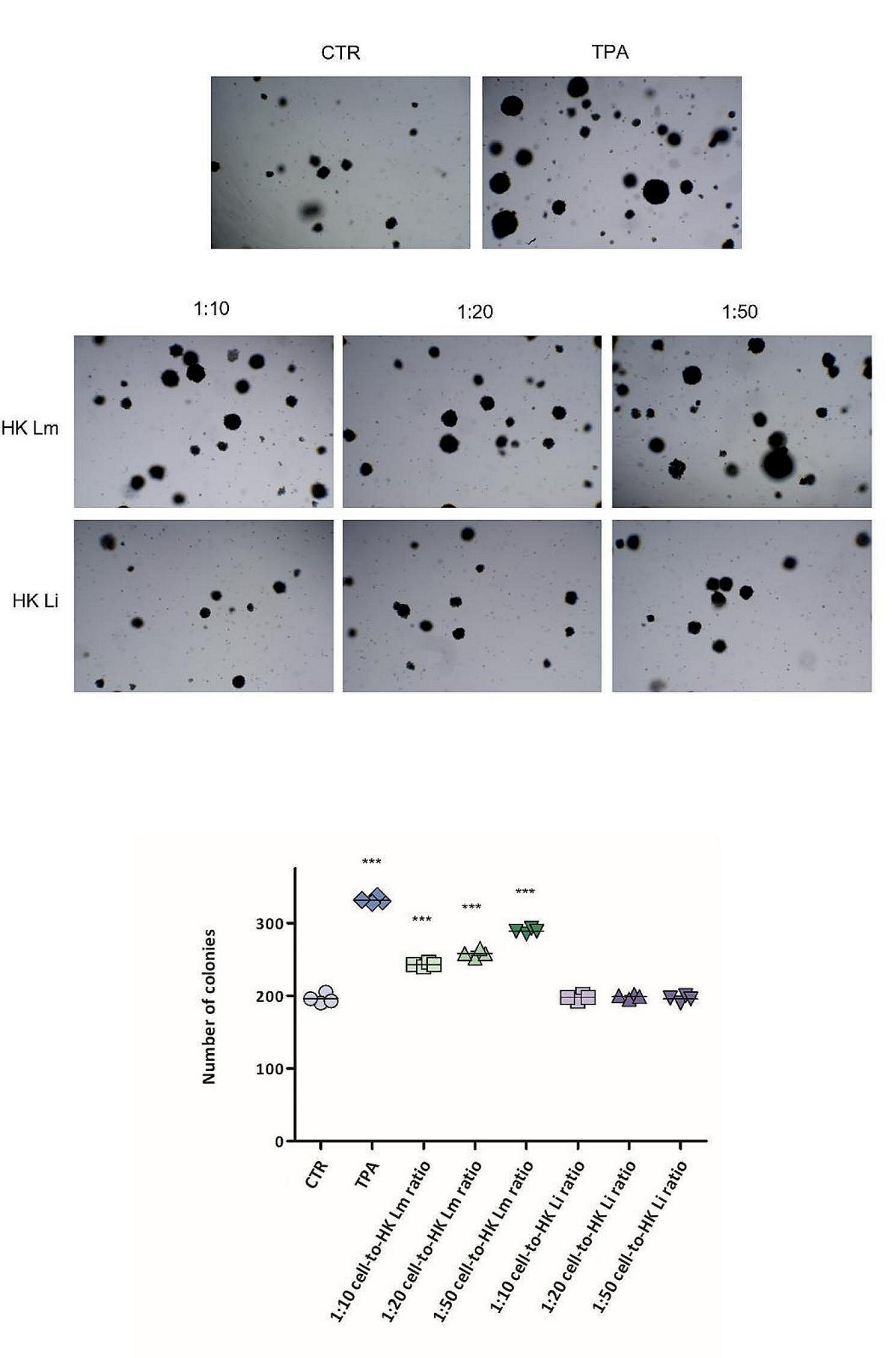
Effects of HK *L. monocytogenes* on JB6 P + cell transformation and tumorigenic capacity in anchorage-independent conditions. Cells were cultured in contact with 1:10, 1:20, or 1:50 cell-to-HK *L. monocytogenes* ratios, or with 1:10, 1:20, or 1:50 cell-to-HK *L. innocua* ratios, in soft agar for 31 days. Representative stereoscope images of 3D colony formation (A); number of colonies formed, after crystal violet staining and stereoscope counting. Data show results of at least three different experiments; results are expressed as the total number of colonies ± standard error (B). Statistical significance is compared to CTR. *** *p* < 0.001; one-way ANOVA, followed by Dunnett’s Multiple Comparison Test. TPA: tumor promoter; HK Lm: heat-killed *L. monocytogenes*; HK Li: heat-killed *L. innocua*

To further investigate the impact of HK *L. monocytogenes* in modulating pre-neoplastic cell transformation, the soft agar assay was performed, with the same cell-to-bacteria ratios (Fig. 2A). As result, the tumorigenic promotion by TPA was confirmed, showing a 1.69- fold increase in cell colony formation capacity in comparison to the control. Moreover, all cell-to-HK *L. monocytogenes* ratios tested led to a statistically significant dose-dependent induction of cell transformation and tumorigenic capacity (1:10 cell-to-HK *L. monocytogenes* ratio: 1.24 ± 0.01; 1:20 cell-to-HK *L. monocytogenes* ratio: 1.32 ± 0.03; 1:50 cell-to-HK *L. monocytogenes* ratio: 1.47 ± 0.02) (Fig. 2B).

Furthermore, the use of suspensions of HK *L. innocua* and inert latex beads, as negative controls, corroborated the specificity of the effects determined by the pathogenic species *L. monocytogenes*. HK *L. innocua* did not change the number of colonies formed in soft agar, in comparison to the control (1:10 cell-to-HK *L. innocua* ratio: 1.01 ± 0.02; 1:20 cell-to-HK *L. innocua* ratio: 1.02 ± 0.03; 1:50 cell-to-HK *L. innocua* ratio: 1.01 ± 0.02) (Fig. 2B), as also the inert latex beads suspensions (Fig. S3).

### Heat-killed *L. monocytogenes* modulates proliferation and microtumor formation of a colorectal cancer cell line

To evaluate the involvement of HK *L. monocytogenes* as a risk factor for tumor promotion, the proliferative capacity of a human colorectal adenocarcinoma cell line (CaCo2) was assessed after contact with suspensions with the same cell-to-bacteria ratios tested in pre-neoplastic cells (1:10, 1:20 or 1:50).

**Fig. 3:**
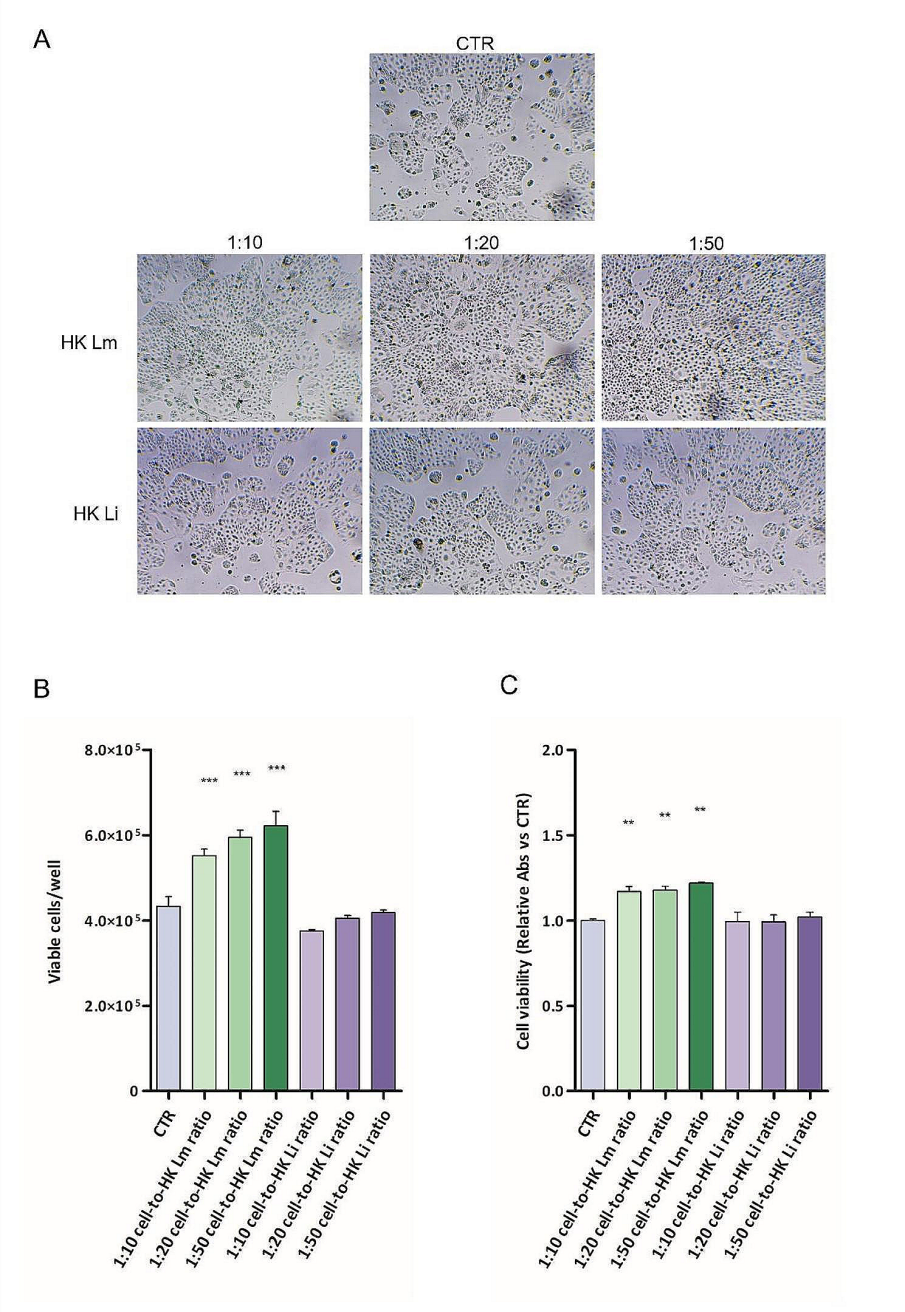
Effects of HK *L. monocytogenes* on CaCo2 cell proliferation in anchorage-dependent conditions. CaCo2 cells were cultured in contact with 1:10, 1:20, or 1:50 cell-to-HK *L. monocytogenes* ratios, or with 1:10, 1:20, or 1:50 cell-to-*L. innocua* ratios, for 48 h. Representative pictures of results (A). Cell viability was assessed through cell count by trypan blue exclusion assay (B) and MTS assay (C). Data show results of at least three different experiments; results are expressed as the relative number of cells (vs. CTR) ± standard error. Statistical significance is compared to CTR. *** *p* < 0.001; one-way ANOVA, followed by Dunnett’s Multiple Comparison Test. HK Lm: heat-killed *L. monocytogenes*; HK Li: heat-killed *L. innocua*

After 48 h of contact, the cancer cell growth was significantly affected by the presence of HK *L. monocytogenes* (Fig. 3A); particularly, the 1:10, 1:20, and 1:50 cell-to-HK *L. monocytogenes* ratios led to a 1.27 ± 0.04, 1.37 ± 0.04, and 1.36 ± 0.10-fold increase in the proliferative potential, respectively. Moreover, in the presence of 1:10, 1:20, and 1:50 cell-to-HK *L. innocua* ratios, the proliferation of cancer cells was not modulated (fold changes: 0.97 ± 0.01, 1.05 ± 0.02, 1.08 ± 0.01, respectively) (Fig. 3B). The same trend of cell viability results was obtained with MTS assay (Fig. 3C).

Even in the presence of inert latex beads suspensions, results were equivalent to those obtained in the control (Fig. S4). All the tested bacterial suspensions did not affect the mortality rate of cancer cells, leading to a number of dead cells comparable to the control cells (Fig. S5).

**Fig. 4:**
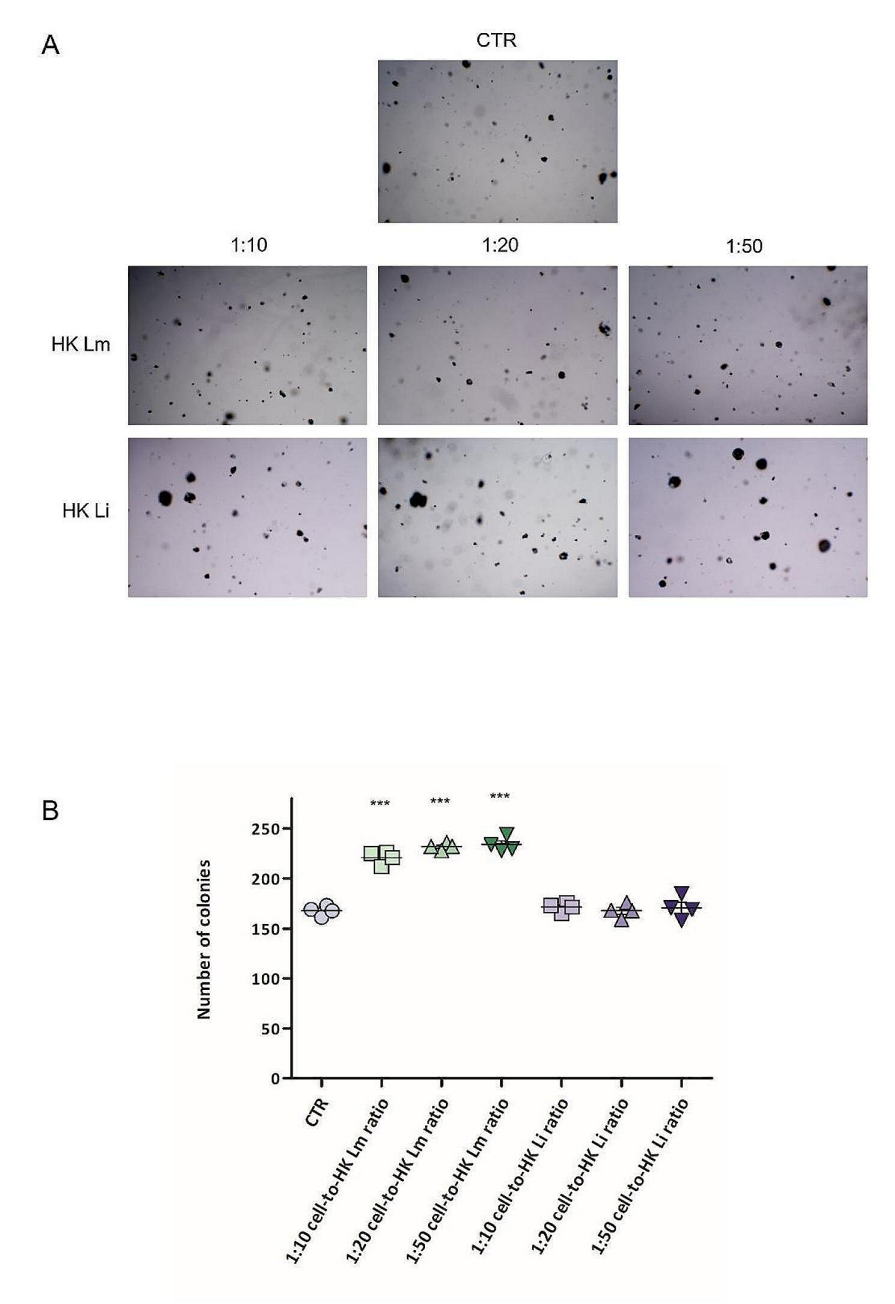
Effects of HK *L. monocytogenes* on CaCo2 cell tumorigenic potential in anchorage-independent conditions. CaCo2 cells were cultured in soft agar layer in contact with 1:10, 1:20, or 1:50 cell-to-HK *L. monocytogenes* ratios, or with 1:10, 1:20 or 1:50 cell-to-HK *L. innocua* ratios, for 31 days. Representative stereoscope images of 3D colony formation (A). Cell tumorigenic potential was assessed by counting the three-dimensional colonies formed. Data show the mean results of four different experiments; results are expressed as the total number of colonies ± standard error (B). Statistical significance is compared to CTR. *** *p* < 0.001; one-way ANOVA, followed by Dunnett’s Multiple Comparison Test. HK Lm: heat-killed *L. monocytogenes*; HK Li: heat-killed *L. innocua*

To further assess the capacity of HK *L. monocytogenes* to modulate the tumorigenic potential of CaCo2 cells, the adenocarcinoma cell line was grown in anchorage-independent conditions in presence of HK *L. monocytogenes* or HK *L. innocua* suspensions, as negative control (Fig. 4A).

As result, HK *L. monocytogenes* induced a significant increase in three-dimensional colony number (Fig. 4B), and data strongly confirm results obtained in anchorage-dependent assay. Particularly, the contact with 1:10, 1:20, and 1:50 cell-to-HK *L. monocytogenes* ratios led to a 1.32 ± 0.05, 1.38 ± 0.02, and 1.40 ± 0.05-fold increase in the tumorigenic potential of cancer cells, in comparison to control one, respectively.

Conversely, neither *L. innocua* nor the inert latex beads affect colony number and the tumorigenic potential of CaCo2 cells (1:10, 1:20, and 1:50 cell-to-HK *L. innocua* ratios led to 1.02 ± 0.03, 1.00 ± 0.05, and 1.02 ± 0.08-fold changes, respectively) (data of inert latex beads available as supplementary material, Fig. S6).

### Involvement of IGF1-R pathway in heat-killed *L. monocytogenes* modulation of cancer cells’ proliferative and tumorigenic capacities

**Fig. 5:**
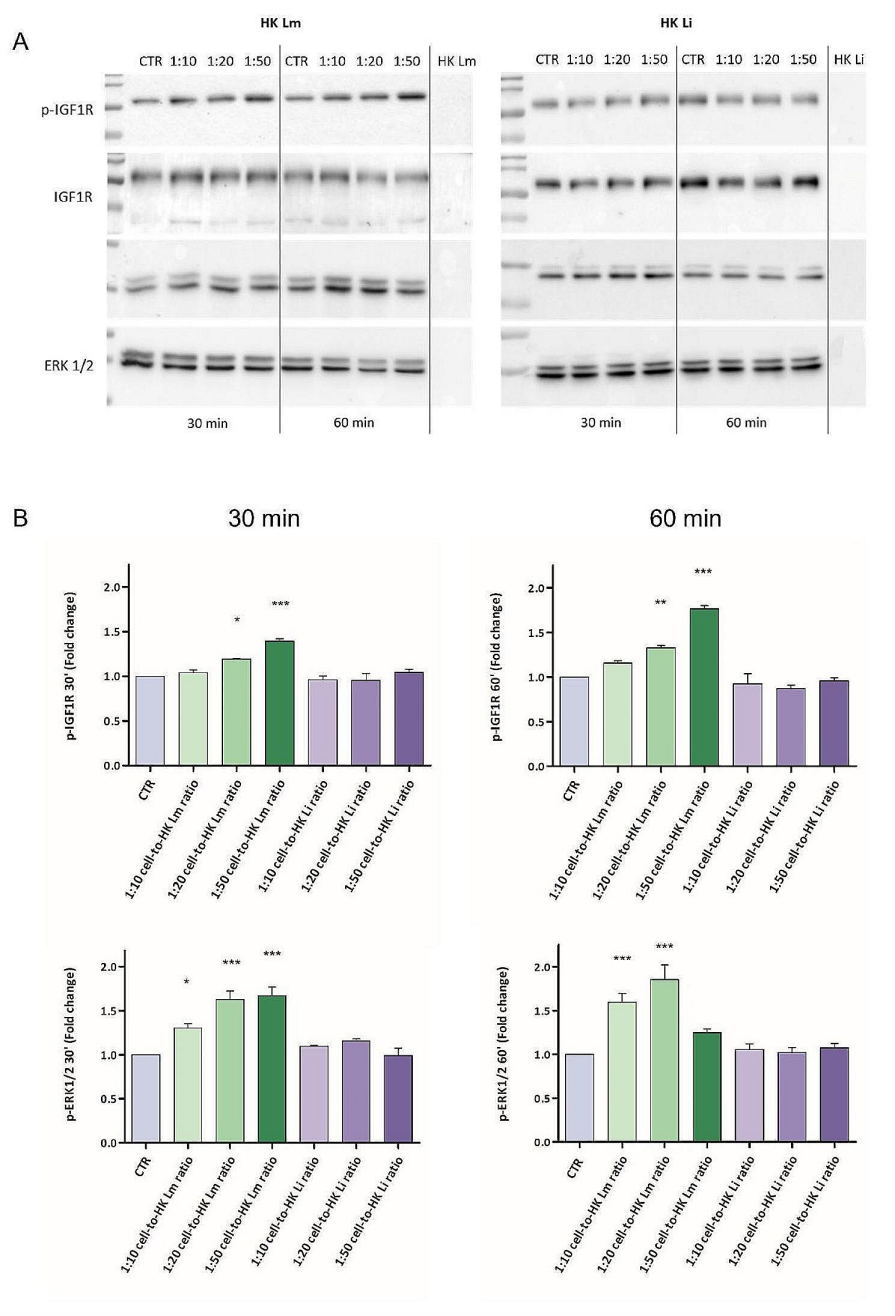
Western Blotting analysis. Representative pictures of the results (A). Blots densitometric analysis and targets quantification (B). Protein samples were obtained by CaCo2 cells cultured in contact with 1:10, 1:20, or 1:50 cell-to-HK *L. monocytogenes* ratios, or with 1:10, 1:20, or 1:50 cell-to-HK *L. innocua* ratios, for 30 or 60 min. Samples of HK *L. monocytogenes* and *L. innocua* were loaded as negative control, too. Results are normalized to the level of total protein of the studied targets. Data show results of at least three different experiments; results are expressed as relative fold change in comparison to the control ± standard error. Statistical significance is compared to CTR. * *p* < 0.05; ** *p* < 0.01; *** *p* < 0.001; one-way ANOVA, followed by Dunnett’s Multiple Comparison Test. Full-length blots are presented in Supplementary Figure S8. HK Lm: heat-killed *L. monocytogenes*; HK Li: heat-killed *L. innocua*

To understand the molecular mechanisms possibly involved in the proliferative and tumorigenic modulation of CaCo2 cells, the expression level of different targets was evaluated, after 30 and 60 min of contact with HK *L. monocytogenes* and HK *L. innocua*, through western blotting analysis (Fig. 5A). The obtained results were normalized to the total protein level of the target studied and data were expressed as relative fold change in comparison to the control.

In particular, the modulation of the phosphorylation levels of IGF-1R and ERK 1/2 was considered. Data showed a dose-dependent significant increase in phospho-IGF-1R levels, in all the cell-to-HK *L. monocytogenes* ratios used, in both the contact times tested (30 or 60 min). Conversely, neither at 30 min nor at 60 min, HK *L. innocua* modulated the protein phosphorylation level in comparison to the control (Fig. 5B).

Moreover, the phosphorylation levels of ERK 1/2 were affected, too. After 30 and 60 min, all the samples with HK *L. monocytogenes* showed an increased phosphorylation level. Also in this case, the phosphorylation of the target protein was not affected by HK *L. innocua* ratios, which led to results equivalent to those obtained in the control samples. Furthermore, samples of HK *L. monocytogenes* and *L. innocua* lysed and loaded in the gels did not give any chemiluminescent signal, confirming that the proteins detected were derived from CaCo2 cells.

Lastly, results similar to HK *L. innocua* were obtained by inert latex beads suspensions, with no change in the tested targets (Fig. S7).

## Discussion

Recent data suggest that infectious pathogens can represent strong and modifiable risk factors in about 15–20% of cancer cases [27]. Although, recently, several studies have provided experimental evidence for an etiological role of bacterial factors disposing infected tissue towards cancer [28], only *H. pylori* is currently accepted as a group 1 carcinogen [19], underlining how the bacteria-cancer field has not received the proper amount of attention, yet [28].

Even though links between cancer induction and bacterial infection exist, it is not clear if genomic instability of cells and increased cell proliferation can be induced by living bacteria, dead bacteria, or by only single components of them [29–32]. Because of the rapid cell-damaging effects of whole living bacterium on eukaryotic cells, in in vitro models the bacterial suspensions are generally inactivated with a thermal treatment; it has been shown that heat-killed (HK) bacteria can cause the immune response and potentiate tumorigenic process in different cancer cell lines [9, 20, 33]. Moreover, the exposure to HK bacteria was shown to trigger substantial molecular responses and cause genomic instability in different organs in mice models, too, and being involved in increased expression of proteins capable of inducing cell proliferation and cancer risk [30, 34].

*L. monocytogenes* is a foodborne pathogen that can cause a severe illness, especially in people with a weakened immune system or comorbidities [35]. Moreover, the presence of *L. monocytogenes* has been shown to be involved in hepatocarcinoma proliferation and progression [20].

In this study, a *L. monocytogenes* strain isolated from ready-to-eat food in Italy was selected to evaluate its tumorigenic potential. As previously shown by Schiavano et al. [22], it belongs to clonal complex 1, which carries two of the hypervirulence pathogenicity islands identified so far (LIPI-1 and LIPI-3) and presents good invasive properties, highlighting its significant health risk for the consumer. Its effects on tumor cell initiation and progression were assessed; for this purpose, the pre-neoplastic cell line JB6 cl 41-5a promotion sensitive (JB6 P+) has been used. The JB6 P + cells are sensitive to TPA stimulation, which induces microtumor formation in soft agar and foci formation in adherent culture conditions, two tumorigenic hallmarks. Our results show a strong induction of cell transformation, proliferation, and tumorigenic process only in the presence of HK *L. monocytogenes* in both anchorage-dependent and independent culture conditions. Conversely, no change was observed in the case of HK *L. innocua* and inert latex beads.

Moreover, the role of *L. monocytogenes* in promoting CRC tumorigenic potential was considered, too, using a human colorectal adenocarcinoma cell line (CaCo2). Also in this case, our results show the effects of the bacteria in enhancing the proliferative and tumorigenic capacities of cancer cells in a significant manner.

Lastly, to evaluate the conceivable mechanism involved, the modulation of the IGF-1R pathway was considered. IGF-1R is a cell membrane receptor and is known to play a critical role in the proliferation cascade, cancer cell development, tumor progression, and resistance to treatments [36]. It has been shown to be highly expressed and activated in the case of *H. pylori*-infected gastric cancer and intestinal metaplasia, suggesting a possible involvement of this pathway in infection-induced cancer proliferation [21]. In agreement with Nakajima et al. [21], we found that *L. monocytogenes* led to an increase in the phosphorylation levels of the receptor and of one of the downstream targets involved in the molecular cascade, suggesting that the bacteria might interact with and activate the receptor. Most importantly, all the noticed results have been suggested as ascribable to the pathogenic strain of *L. monocytogenes*, because of the absence of modulative effects in proliferative capacity, tumorigenic ability, and molecular targets considered with the non-pathogenic strain *L. innocua*, used as a negative control.

The potential limitation of the present work is represented by the in vitro experimental settings. However, the three-dimensional approach proposed is more accurate in reflecting the natural tumor microenvironment with respect to the anchorage-dependent experiments [37], being considered an important step before performing animal studies. Further studies will aim to confirm the role of the IGF1R pathway as a biological mechanism involved in the observed effects; to this end, the use of specific inhibitors of the receptor or the development of a IGF1R knockdown cell line will be evaluated.

## Conclusion

Our data suggest that the prevention of the foodborne pathogen *L. monocytogenes* infections is essential, with particular attention to cancer patients. Moreover, it paves the way for the detection of bacterial infiltration in tumors and possible further antibiotic therapy as essential steps for cancer progression control. Indeed, we can assume the selective elimination of the pathogenic bacteria in tumor niche or pre-neoplastic cells could be a prevention method to reduce the risk of initiation, progression, and tumor recurrence. Yet, there is still a lack of deeper understanding of how these bacteria may be associated with the cause of cancer. This highlights the need for more research to be done in this field.

### Electronic supplementary material

Below is the link to the electronic supplementary material.

Supplementary Material 1

## Data Availability

Data are included in article and supplementary material files. The datasets used and/or analyzed during the current study are available from the corresponding author on reasonable request.
