# Supplementary material for: The potential role of Listeria monocytogenes in promoting colorectal adenocarcinoma tumorigenic process
Source: BMC Microbiol. 2024 Mar 15;24:87. doi: 10.1186/s12866-024-03240-5 (PMC10941472; doi:10.1186/s12866-024-03240-5)
Supplement: Supplementary file 1 — Supplementary Material 1 [file 12866_2024_3240_MOESM1_ESM.docx]

**Supplementary Figures**

**
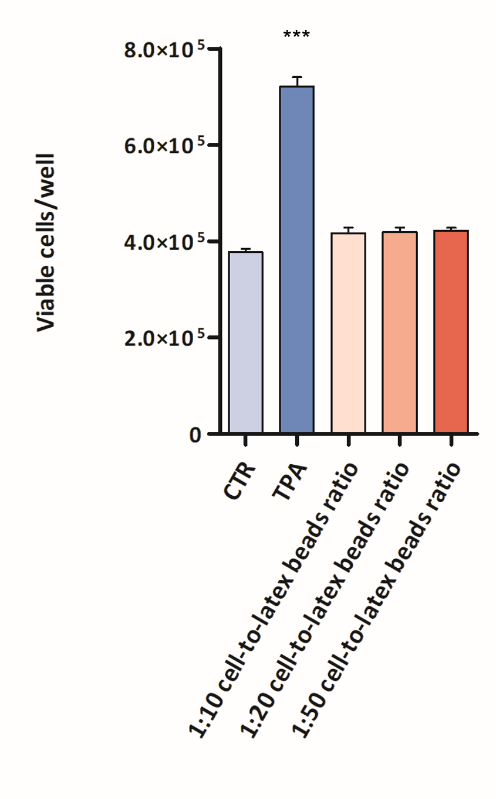
**

**Fig S1.** Effects of inert latex beads on JB6 P+ cell transformation and proliferation in anchorage-dependent conditions. Cells were cultured in contact with 1:10, 1:20 or 1:50 cell-to-latex beads ratios, for 72 hours. Cell viability was assessed through trypan blue exclusion assay. Data show results of at least three different experiments; results are expressed as total number of cells ± standard error. Statistical significance is compared to CTR. *** p<0.001; one-way ANOVA, followed by Dunnett's Multiple Comparison Test.
TPA: tumor promoter.


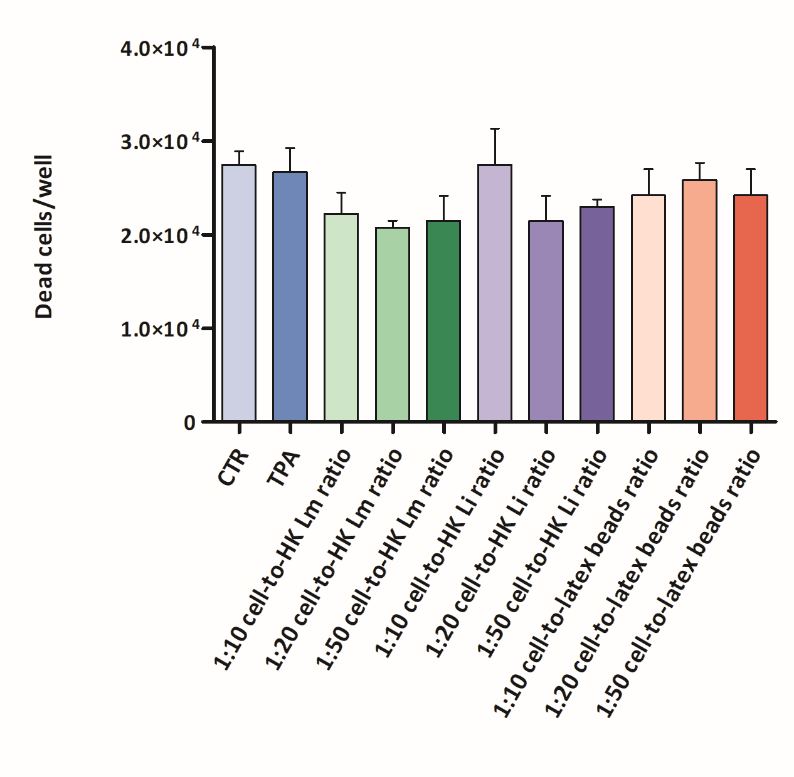


**Fig. S2:** Effects of HK *L. monocytogenes* on JB6 P+ mortality in anchorage-dependent conditions. Cells were cultured in contact with 1:10, 1:20 or 1:50 cell-to-HK *L. monocytogenes*, cell-to-HK *L. innocua*, or cell-to-latex beads ratios for 72 hours. Cell death was assessed through trypan blue exclusion assay. Data show results of at least three different experiments; results are expressed as total number of cells ± standard error. Statistical analysis is compared to the CTR; one-way ANOVA, followed by Dunnett's Multiple Comparison Test.
TPA: tumor promoter; HK Lm: heat-killed *L. monocytogenes*; HK Li: heat-killed *L. innocua*.

**
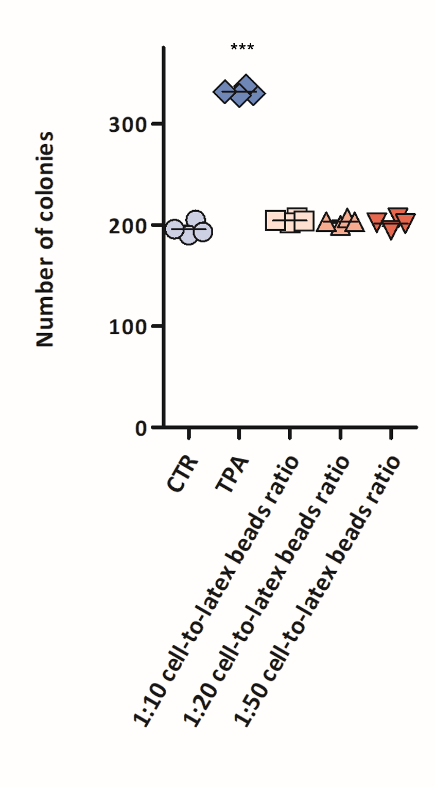
**

**Fig. S3:** Effects of inert latex beads on JB6 P+ cell transformation and tumorigenic capacity in anchorage-independent conditions. Cells were cultured in contact with 1:10, 1:20 or 1:50 cell-to-latex beads ratios, in soft agar for 31 days. Number of colonies formed, after crystal violet staining and stereoscope counting. Data show results of at least three different experiments; results are expressed as total number of colonies ± standard error. Statistical significance is compared to CTR. *** p<0.001; one-way ANOVA, followed by Dunnett's Multiple Comparison Test.
TPA: tumor promoter.

**
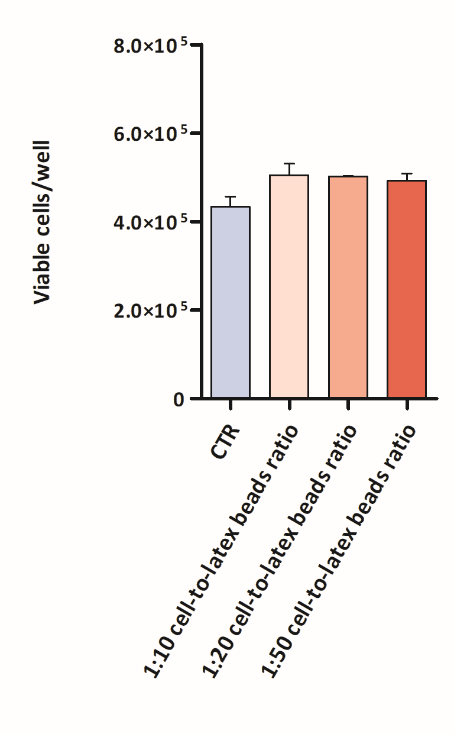
**

**Fig S4.** Effects of inert latex beads on CaCo2 cell transformation and proliferation in anchorage-dependent conditions. Cells were cultured in contact with 1:10, 1:20 or 1:50 cell-to-latex beads ratios, for 72 hours. Cell viability was assessed through trypan blue exclusion assay. Data show results of at least three different experiments; results are expressed as total number of cells ± standard error. Statistical significance is compared to CTR; one-way ANOVA, followed by Dunnett's Multiple Comparison Test.


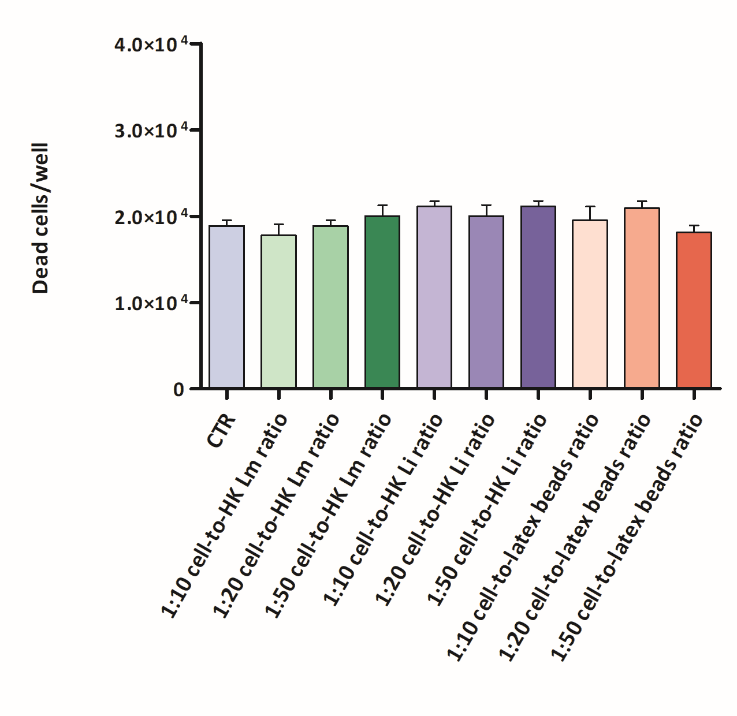


**Fig. S5:** Effects of HK *L. monocytogenes* on CaCo2 mortality in anchorage-dependent conditions. Cells were cultured in contact with 1:10, 1:20 or 1:50 cell-to-HK *L. monocytogenes*, cell-to-HK *L. innocua*, or cell-to-latex beads ratios for 48 hours. Cell death was assessed through trypan blue exclusion assay. Data show results of at least three different experiments; results are expressed as total number of cells ± standard error. Statistical significance is compared to CTR; one-way ANOVA, followed by Dunnett's Multiple Comparison Test.

HK Lm: heat-killed *L. monocytogenes*; HK Li: heat-killed *L. innocua.*

**
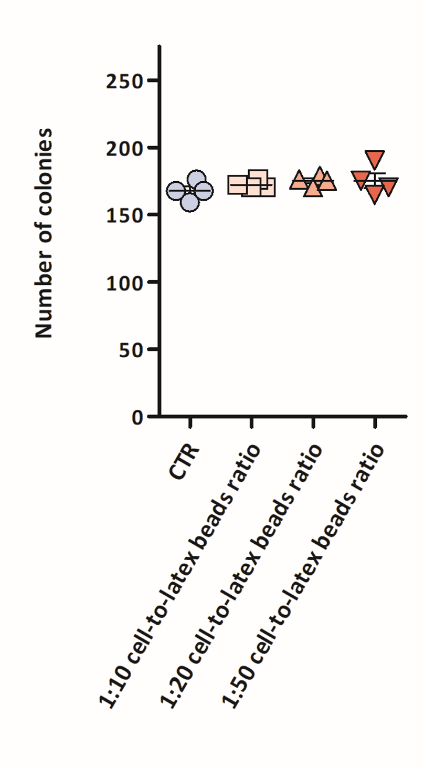
**

**Fig. S6:** Effects of inert latex beads on CaCo2 cell transformation and tumorigenic capacity in anchorage-independent conditions. Cells were cultured in contact with 1:10, 1:20 or 1:50 cell-to-latex beads ratios, in soft agar for 31 days. Number of colonies formed, after crystal violet staining and stereoscope counting. Data show results of at least three different experiments; results are expressed as total number of colonies ± standard error. Statistical significance is compared to CTR; one-way ANOVA, followed by Dunnett's Multiple Comparison Test.

**
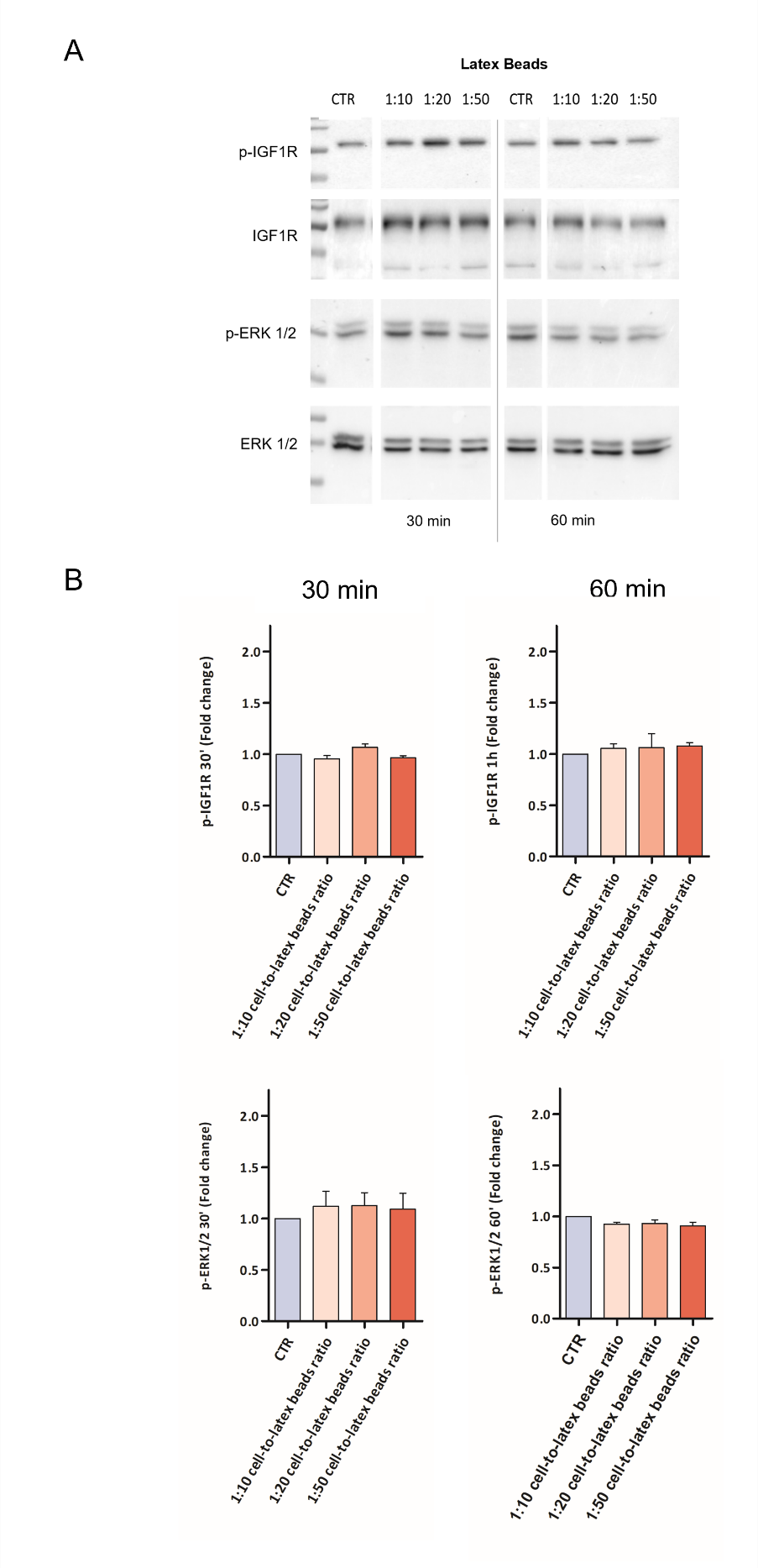
**

**Fig S7.** Western Blotting analysis. Representative pictures of the results (A). Targets quantification (B). Protein samples were obtained by CaCo2 cells cultured in contact with 1:10, 1:20 or 1:50 cell-to-latex beads ratios, for 30 or 60 minutes. Results are normalized to level of total protein of the studied targets. Data show results of at least three different experiments; results are expressed as relative fold change in comparison to the control ± standard error. Statistical significance is compared to CTR; one-way ANOVA, followed by Dunnett's Multiple Comparison Test.


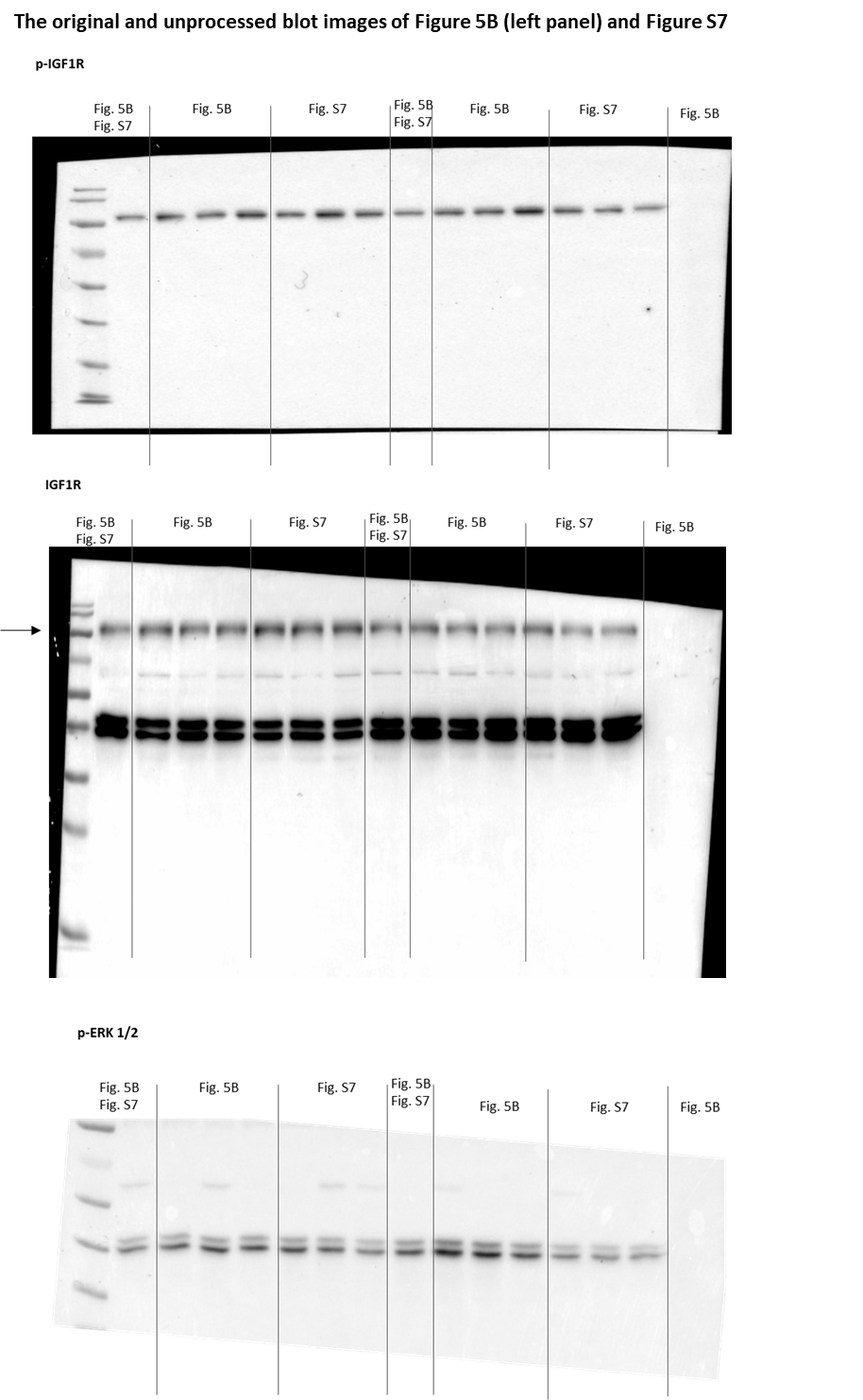


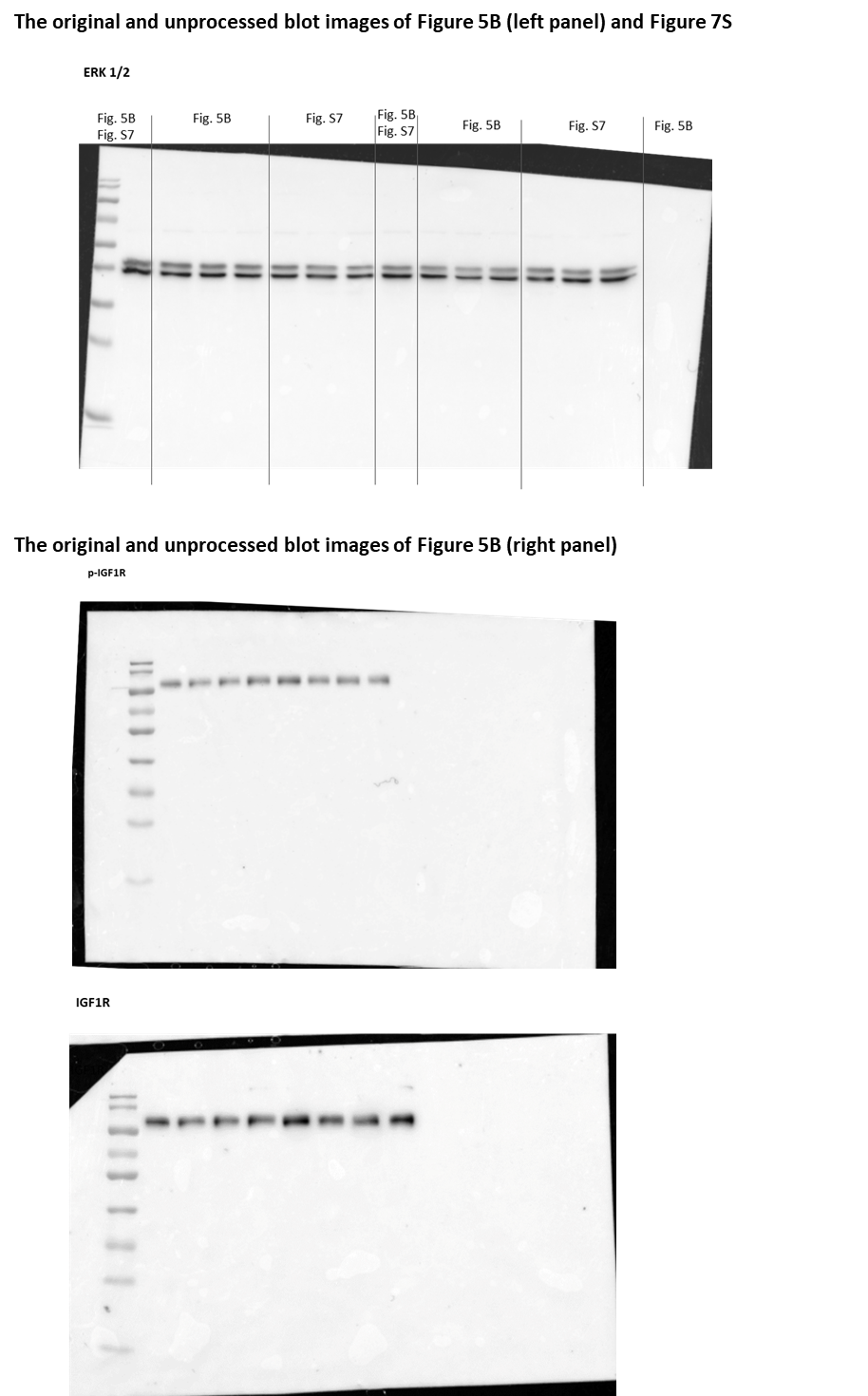


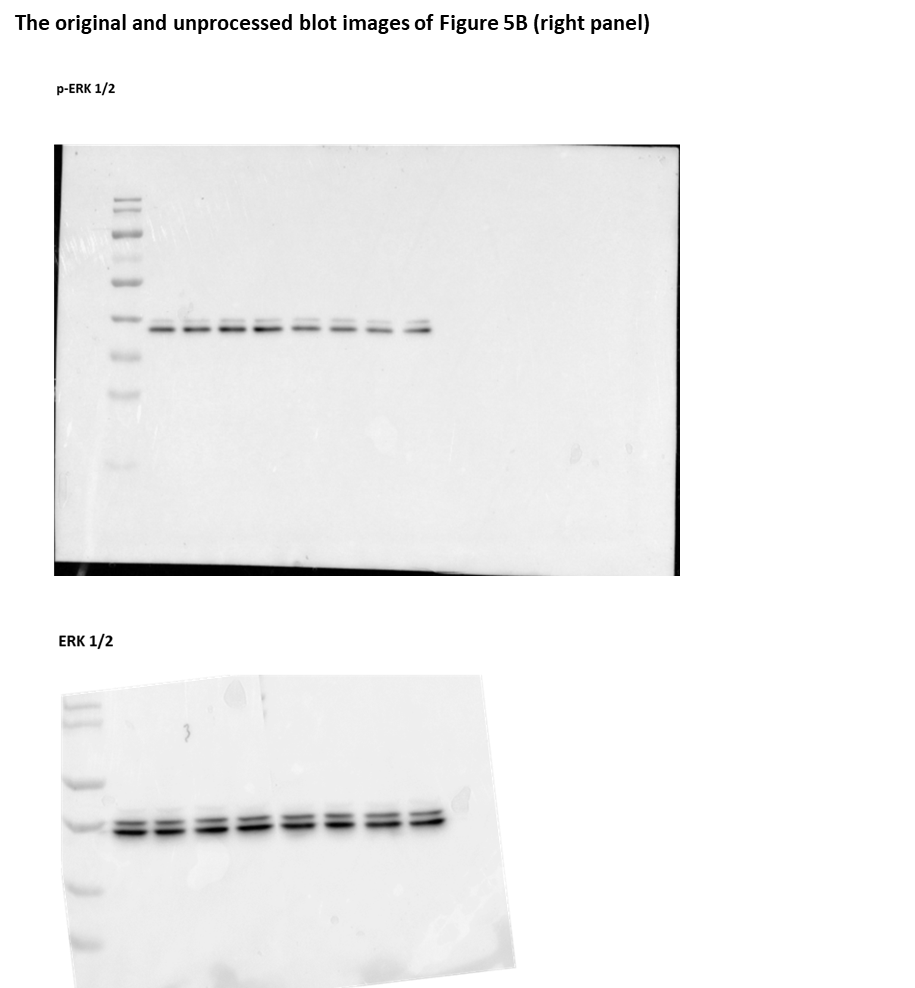


**Fig S8**. Full-length blots of Western Blotting analysis.
